# A national survey of the diagnosis and management of Ménière’s disease among ENT consultants, UK

**DOI:** 10.1308/rcsann.2025.0028

**Published:** 2025-06-17

**Authors:** S Parihar, FS Koumpa, C Neumann, RG Kanegaonkar

**Affiliations:** ^1^ University Hospitals Sussex NHS Foundation Trust, UK; ^2^ Barts Health NHS Trust, UK; ^3^ East Kent Hospitals University NHS Foundation Trust, UK; ^4^ Medway NHS Foundation Trust, UK; ^5^ Institute of Medical Sciences, Canterbury Christ Church University, UK

**Keywords:** Ménière’s disease, Diagnosis, ENT consultants, UK, Survey

## Abstract

**Introduction:**

Ménière's disease (MD) is a rare condition whose diagnosis can be challenging. The American Academy of Otorhinolaryngology-Head and Neck Surgeons (AAO-HNS) has published new guidance to facilitate the diagnosis of MD. We surveyed ear, nose and throat (ENT) consultants in the United Kingdom (UK) to assess their confidence in diagnosing MD, their use of the AAO-HNS guidance and current diagnostic and treatment approaches.

**Methods:**

An online questionnaire was distributed. It asked respondents to anonymously rank their confidence in diagnosing MD, identify the minimum investigations required to make a diagnosis, describe their use of the AAO-HNS criteria, share their preferred treatment for acute attacks and state first- and second-line preventative treatment options.

**Results:**

A total of 86 responses were collected. In total, 88% of respondents reported high levels of confidence in diagnosing MD. Most respondents (29.1%) stated the minimum tests required were ‘history’, ‘otoscopy’, ‘pure tone audiometry’ and ‘MRI’ (magnetic resonance imaging), although some chose as few as one test (3.49%) and others up to seven (1.2%). Regarding use of the AAO-HNS criteria, responses ranged from ‘always’ (34.9%) to ‘never’ (20.9%). Prochlorperazine was the first-line treatment for acute attacks for 81.4% of respondents. Betahistine (38.4%) and dietary restrictions (37.2%) were recommended almost equally as first-line preventative measures. The most popular second-line measure was intratympanic steroids (34.9%), followed by betahistine (24.4%).

**Conclusion:**

Our survey revealed wide disparities in the diagnosis and management of MD by ENT consultants in the UK, and AAO-HNS guidelines were not universally used. We propose developing greater consensus and intend to conduct a similar international survey to gather a broader perspective.

## Introduction

Ménière’s disease (MD) is a rare condition that results in spontaneous, severe bouts of rotatory vertigo, fluctuating sensorineural hearing loss and roaring tinnitus. This debilitating condition can significantly impact the quality of life of patients and their families.

Despite ongoing research, the underlying cause of MD remains elusive. Both cadaveric and, more recently, gadolinium-enhanced magnetic resonance imaging (MRI) studies have demonstrated expansion of the scala media and endolymphatic compartments.^1,2^ The cause of this volume change has been attributed to either an abnormal overproduction or reduced resorption of endolymph. A variety of aetiologies have been proposed including vascular, autonomic, viral, allergic, genetic and autoimmune.^5–10^

The diagnosis of MD can be challenging because episodes are intermittent, and attacks can occur weeks or months apart. The American Academy of Otorhinolaryngology-Head and Neck Surgeons (AAO-HNS) criteria are often used in the diagnosis of this condition and published clinical trials have adhered to their classification. However, although recently updated, the criteria for ‘definite MD’ and ‘probable MD’ are not widely used, with anecdotal evidence suggesting that clinicians apply either softer or more stringent criteria to establish a diagnosis.^11^ Unsurprisingly, treatment of this condition is also variable with some advocating lifestyle changes, medical therapy, function-preserving or destructive procedures, or surgical intervention.

As a result of the controversies surrounding the diagnosis and treatment of MD, we conducted a survey to assess the confidence level, minimum diagnostic data and initial and secondary management of this condition among consultant ear, nose and throat surgeons (ENT) in the United Kingdom (UK).

## Methods

An anonymous online questionnaire, approved by the Research and Innovation Department of Medway Maritime Hospital, Gillingham, UK, was generated via Google Forms^®^ and distributed electronically to consultant ENT surgeons in the UK (Appendix 1 – available online). The consultants included in our study were specifically chosen based on their subspecialty interest in otology from various regions across the UK. To ensure broad representation, the survey was initially distributed through otology networks, encompassing major centres throughout the country. Subsequently, participants were encouraged to share the survey further within their networks.

The questionnaire first asked respondents to rank their confidence in diagnosing MD on a scale of 1 (not confident) to 5 (very confident). Respondents were also asked to identify the minimum required investigations for a diagnosis of MD from a list of 11 items that ranged from clinical assessment to radiological imaging and formal vestibular tests. In addition, the survey assessed use of the AAO-HNS guidelines in diagnosing MD and the preferred first-line treatment for acute attacks of the disease. Finally, respondents were asked to choose their first-line treatment option to prevent acute attacks from a list of 16 items (with an option to include treatments not listed) and their second-line option should this fail.

The survey accepted responses submitted during a 4-week period from 16 March to 16 April 2023.

## Results

**Figure 1:**
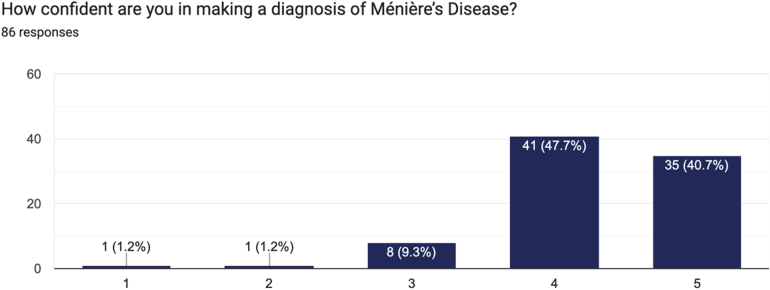
Survey responses question 1

A total of 86 responses were recorded. On asking clinicians about their confidence in diagnosing MD, the majority of respondents reported high levels of confidence. On a scale of 1 (not confident) to 5 (very confident), 47.7% (*n *= 41) reported a score of 4 and 40.7% (*n *= 35) reported a score of 5 (Figure 1).

**Figure 2:**
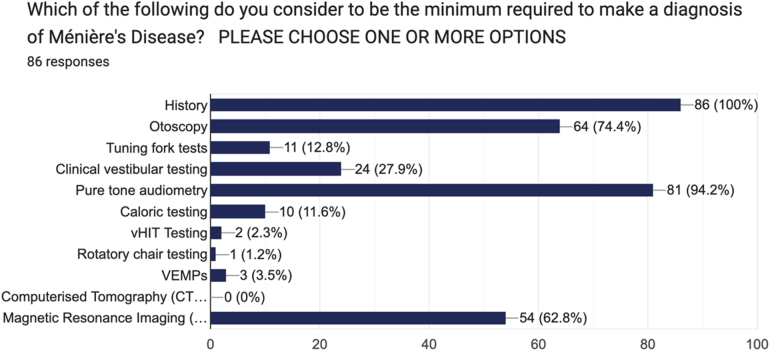
Survey responses question 2

The second question asked clinicians about the minimum tests required to make a diagnosis of MD. Respondents were asked to choose 1 or more responses from an 11-item list ranging from simple history and examination to clinical vestibular testing, caloric testing and MRI. The results were highly varied from ‘history’ alone (*n *= 3) to ‘history, otoscopy, tuning fork tests, clinical vestibular testing, pure tone audiometry, caloric testing and video head impulse testing (vHIT)’ (*n *= 1). The majority of respondents (29%) stated that ‘history, otoscopy, pure tone audiometry and MRI’ (*n *= 25) would be the minimum tests required (Figure 2).

**Figure 3:**
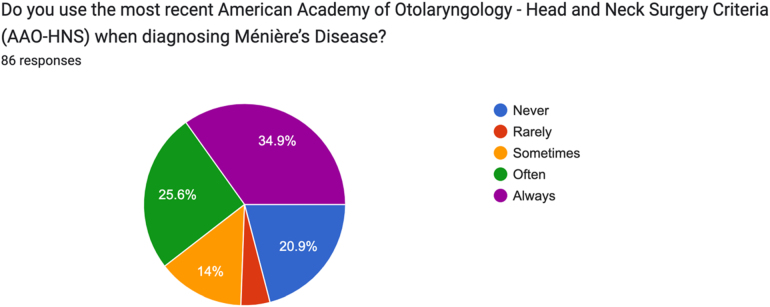
Survey responses question 3

Responses varied significantly when respondents were asked whether they used the most recent AAO-HNS criteria for diagnosing MD. Answers ranged from ‘always’ (34.9%, *n *= 30) to ‘never’ (20.9%, *n *= 18). Of the remainder, 25.6% (*n *= 22) stated that they used the criteria ‘often’, 14.0% (*n *= 12) ‘sometimes’ and 4.7% (*n *= 4) ‘rarely’ (Figure 3).

**Figure 4:**
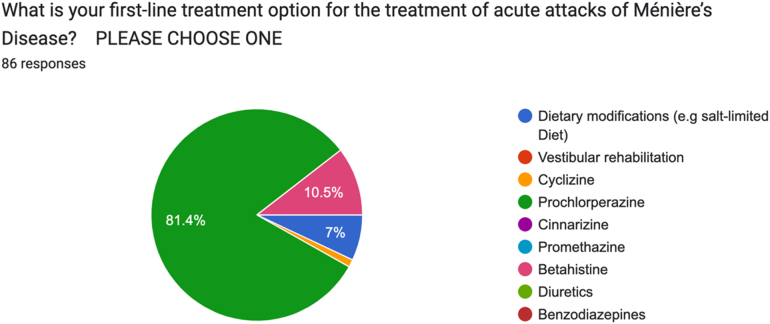
Survey responses question 4

The first-line treatment recommended for acute attacks in the majority of patients (81.4%, *n *= 70) was prochlorperazine. Respondents also recommended betahistine (10.5%, *n *= 9), dietary restrictions (7.0%, *n *= 6) and cyclizine (1.2%, *n *= 1) (Figure 4). Diuretics, benzodiazepines, promethazine, cinnarizine and vestibular rehabilitation were not chosen.

With regards to the first-line preventative measure recommended, the majority of respondents were split almost equally between the use of ‘betahistine’ (38.4%, *n *= 33) and ‘dietary restrictions (e.g. salt-limiting diet)’ (37.2%, *n *= 32). The remainder were split between intratympanic steroids (8.1%, *n *= 7), grommet insertion with intratympanic steroid injection (5.8%, *n *= 5), vestibular rehabilitation (3.5%, *n *= 3), diuretics (2.3%, *n *= 2), cinnarizine (1.2% *n *= 1), lifestyle modification (1.2%, *n *= 1) and two free-text responses indicating a combination of dietary modifications and medical management (2.4%, *n *= 2). Surgical labyrinthectomy, gentamicin ablation, intratympanic gentamicin, vestibular nerve section, saccus decompression, grommet insertion with gentamicin, grommet insertion and prochlorperazine were not selected.

**Figure 5:**
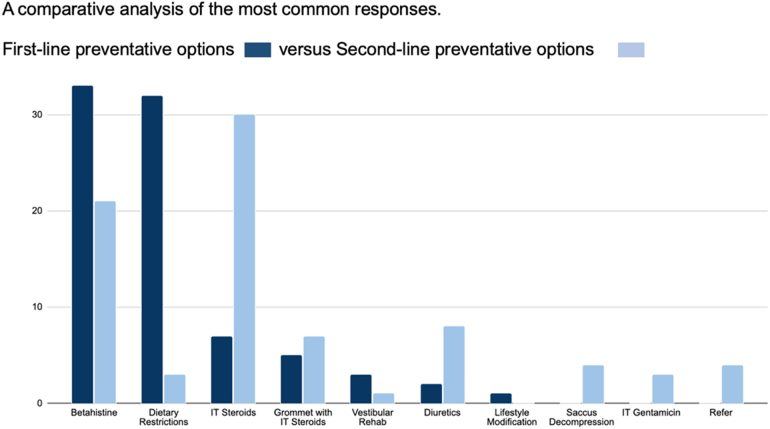
Comparative analysis of the most common responses to questions 5 and 6

The recommended second-line preventative measure if the first-line option were to fail also displayed substantial variability. The most popular choice was ‘intratympanic steroids’ (34.9%, *n *= 30) followed by ‘betahistine’ (24.4%, *n *= 21) and ‘diuretics’ (9.3%, *n *= 8). A minority selected grommet insertion with intratympanic steroid injection (8.1%, *n *= 7), saccus decompression (4.7%, *n *= 4), dietary restrictions (3.5%, *n *= 3) and intratympanic gentamicin (3.5% *n *= 3). One response was given to each of vestibular rehabilitation, grommet insertion, anti-migraine medication and endolymphatic duct ligation (1.2%, *n *= 1 each). Four respondents used the free-text insertion to state that they would refer the patient to a balance expert (1.2%, *n *= 1), colleague (1.2%, *n *= 1), specialist otologist (1.2%, *n *= 1) and otologist (1.2%, *n *= 1). Two doctors felt they were unable to respond to the question (*n *= 2). Surgical labyrinthectomy, gentamicin ablation, vestibular nerve section, grommet insertion with intratympanic gentamicin injection, promethazine, cinnarizine, prochlorperazine, diuretics, dietary restrictions (e.g. salt-limiting diet) and betahistine were not chosen (Figure 5).

## Discussion

MD is a disorder of the inner ear that results in symptoms of spontaneous vertigo, aural pressure, tinnitus and hearing loss. Because of its intermittent and unpredictable course, treatment outcomes may be difficult to assess.

Episodes classically result in an initial fluctuating sensorineural hearing loss that gradually declines and plateaus to 60dB hearing level. Episodes are also associated with a decline in peripheral vestibular function. This can be seen as abnormal vestibular function tests including calorics, vHIT and nystagmography, but atypical presentations with normal vestibular function tests do exist.^12–15^

The majority of UK respondents reported high confidence in diagnosing MD, but only 60.5% reported regular use of the new AAO-HNS criteria. The AAO-HNS criteria, first published in 1995, were updated in 2015 attempting to re-classify the condition into ‘definite MD’ and ‘probable MD’.^11^ The varied results of our survey demonstrate a lack of confidence or lack of awareness of the AAO-HNS criteria in our cohort. Previously published papers may be challenging to navigate because of the evolving diagnostic criteria. In addition, newly published papers describing the outcomes of interventions may be limited in their clinical application and provide data of little practical value in the long term.

Patients who develop any form of hearing loss may have the sensation of aural fullness. If such patients were to develop dizziness they may be incorrectly, although understandably, mislabelled as having developed MD. Hence, the incidence of true MD may be lower than the generally quoted 3.5–513 per 100,000 population, especially if patients are diagnosed in general practice where audiovestibular and imaging services may be unavailable.^16^

Several recently published Cochrane reviews have evaluated various interventions for MD, including diuretics, betahistine, intratympanic dexamethasone injections, intratympanic gentamicin and endolymphatic sac surgery.^17–21^ The reviews relied on high-quality large randomised controlled trials and often concluded that there was insufficient evidence to support any of these interventions in the management of MD.

It may be appropriate, therefore, in this situation to exploit papers of lower-order levels of evidence in the management of MD. Treatments such as grommet insertion with or without steroid administration, endolymphatic sac shunt and tenotomy of the middle ear muscles have been looked at in smaller numbers with good outcomes.^22–32^ Intratympanic methylprednisolone was also compared with gentamicin and shown to provide similar long-term vertigo control without the ototoxicity risk.^33,34^ There is value in further exploring such treatment options, because they have a lower side effect profile and promising results.

Our survey demonstrated that most clinicians recommend intratympanic steroid injections as the first-line treatment of MD. This is seen as an option, but not a recommendation in the new AAO-HNS guidance. Intratympanic gentamicin is recommended when non-ablative treatment has failed, but is not that widely used in the UK.

In the UK, it is standard practice to obtain a pure tone audiogram prior to a patient undergoing ear surgery as this allows the clinician to assess the hearing thresholds in the affected ear and the function of the contralateral ear. Interestingly, however, it is clear from the survey that many ENT surgeons in the UK do not formally assess the vestibular function of the affected ear, let alone the contralateral one, before initiating MD treatment. Neither is this recommended by AAO-HNS guidance. This can have significant implications for the overall patient outcomes. It may be the case that ablative and destructive treatments risk early multilevel vestibulopathy, while patients with an undiagnosed contralateral hypofunction may be left with bilateral vestibular hypofunction, which would render them wheelchair-bound with very limited movement and a very poor quality of life. The authors therefore recommend that patients undergo formal vestibular testing to assess function in the non-affected ear before starting toxic treatment, but in general, that destructive interventions be avoided.

## Study limitations

The study collected responses from 86 participants, representing only a limited proportion of national practice. It also did not capture information on the respondents’ levels of experience in managing MD, and it should be noted that not all participants routinely encounter these patients in their regular clinical practice. Furthermore, the availability of certain investigations, particularly formal vestibular testing, varies across different units in the UK, which may have influenced some respondents’ choices. The survey did not explore the underlying reasons behind each response, and therefore could not assess the attitudes of participants towards certain topics such as the AAO-HNS criteria or acute and preventative interventions. Finally, some respondents found the options provided in the survey too simplistic and felt they did not account for all the components that influence their decision making process. Thus, future studies may consider expanding on the options available for MD management or using a different survey format to account for more components of clinical decision making.

## Conclusion

The results of our survey have provided a snapshot of MD diagnoses and management across the UK. Interestingly, they have demonstrated significant variation in national practice both in acute and preventative treatment. This disparity suggests an urgent need for updated criteria and stricter guidelines to avoid increasing variability in patient journeys, especially considering the significant impact this condition can have on patients’ quality of life.

To gain a more comprehensive understanding of global practices, we aim to replicate this survey on an international level. By collecting data from a broader range of healthcare professionals, we hope to gain insights into the similarities and differences between countries that may inform the development of international guidelines.

## References

[1] Carey JP, Minor LB, Nager GT. Dehiscence or thinning of bone overlying the superior semicircular canal in a temporal bone survey. *Arch Otolaryngol Head Neck Surg* 2000; **126**: 137. 10680863 10.1001/archotol.126.2.137

[2] Lida T, Teranishi M, Yoshida T *et al.* Magnetic resonance imaging of the inner ear after both intratympanic and intravenous gadolinium injections. *Acta Otolaryngol* 2013; **133**: 434–438. 23294239 10.3109/00016489.2012.753640

[3] Hallpike CS, Cairns H. Observations on the pathology of Ménière’s syndrome. *Proc R Soc Med* 1938; **31**: 1317–1336. 19991672 10.1177/003591573803101112 PMC2076781

[4] Yamakawa K. The pathology of a labyrinth with Meniere’s disease. *Jpn J Otol* 1938; **44**: 2310–2312.

[5] Miller JM, Ren TY, Nuttall AL. Studies of inner ear blood flow in animals and human beings. *Otolaryngol Head Neck Surg* 1995; **112**: 101–113. 7816443 10.1016/S0194-59989570308-X

[6] Ishii M, Ishiyama G, Ishiyama A *et al.* Relationship between the onset of Ménière’s disease and sympathetic hyperactivity. *Front Neurol* 2022; **13**: 1–12. 10.3389/fneur.2022.804777 PMC8970286 35370896

[7] Gacek RR. Ménière’s disease is a viral neuropathy. *ORL J Otorhinolaryngol Relat Spec* 2009; **71**: 78–86. 19142031 10.1159/000189783

[8] Banks C, McGinness S, Harvey R, Sacks R. Is allergy related to Meniere’s disease? *Curr Allergy Asthma Rep* 2012; **12**: 255–260. 22457225 10.1007/s11882-012-0258-3

[9] Chiarella G, Petrolo C, Cassandro E. The genetics of Ménière’s disease. *Appl Clin Genet* 2015; **8**: 9–17. 25609993 10.2147/TACG.S59024 PMC4293923

[10] Greco A, Gallo A, Fusconi M *et al.* Meniere’s disease might be an autoimmune condition? *Autoimmun Rev* 2012; **11**: 731–738. 22306860 10.1016/j.autrev.2012.01.004

[11] Lopez-Escamez J, Carey J, Chung W-H *et al.* Diagnostic criteria for Meniere’s disease. Consensus document of the Bárány Society, the Japan Society for Equilibrium Research, the European Academy of Otology and Neurotology (EAONO), the American Academy of Otolaryngology-Head and Neck Surgery (AAO-HNS) and the Korean Balance Society. *Acta Otorrinolaringol Esp* 2015; **67**: 1–7. 26277738 10.1016/j.otorri.2015.05.005

[12] Jerin C, Maxwell R, Gürkov R. High-Frequency horizontal semicircular canal function in certain Menière’s disease. *Ear & Hearing* 2019; **40**: 128–134. 29762197 10.1097/AUD.0000000000000600

[13] Limviriyakul S, Luangsawang C, Suvansit K *et al.* Video head impulse test and caloric test in definite Ménière’s disease. *Eur Arch Otorhinolaryngol* 2020; **277**: 679–686. 31749057 10.1007/s00405-019-05735-8

[14] Rosengren SM, Young AS, Taylor RL, Welgampola MS. Vestibular function testing in the 21st century: video head impulse test, vestibular evoked myogenic potential, video nystagmography; which tests will provide answers? *Curr Opin Neurol* 2022; **35**: 64–74. 34889807 10.1097/WCO.0000000000001023

[15] Hannigan IP, Rosengren SM, Young AS *et al.* A portrait of Menière’s disease using contemporary hearing and balance tests. *Otol Neurotol* 2022; **43**: e489–e496. 35085109 10.1097/MAO.0000000000003479

[16] Alexander TH, Harris JP. Current epidemiology of Meniere’s syndrome. *Otolaryngol Clin North Am* 2010; **43**: 965–970. 20713236 10.1016/j.otc.2010.05.001

[17] Thirlwall AS, Kundu S. Diuretics for Ménière’s disease or syndrome. *Cochrane Database Syst Rev* 2006; **2006**: CD003599. 16856015 10.1002/14651858.CD003599.pub2 PMC9007146

[18] James AL, Burton MJ. Betahistine for Menière’s disease or syndrome. *Cochrane Database Syst Rev* 2001; **2001**: CD001873. 11279734 10.1002/14651858.CD001873 PMC6769057

[19] Phillips JS, Westerberg B. Intratympanic steroids for Ménière’s disease or syndrome. *Cochrane Database Syst Rev* 2011: CD008514. 21735432 10.1002/14651858.CD008514.pub2 PMC13378880

[20] Pullens B, van Benthem PP. Intratympanic gentamicin for Ménière’s disease or syndrome. *Cochrane Database Syst Rev* 2011: CD008234. 21412917 10.1002/14651858.CD008234.pub2 PMC13378876

[21] Pullens B, Giard JL, Verschuur HP etal. Surgery for Ménière’s disease. *Cochrane Database Syst Rev* 2010: CD005395. 20091573 10.1002/14651858.CD005395.pub2

[22] Kanegaonkar R, Najuko-Mafemera A, Hone R, Tikka T. Menière’s disease treated by grommet insertion. *Ann R Coll Surg Engl* 2019; **101**: 602–605. 31508988 10.1308/rcsann.2019.0099 PMC6818060

[23] Kim CS, Martinez U, Mulvey E *et al.* Outcomes of transtympanic dexamethasone perfusion using the MicroWickTM in patients with Ménière’s disease: a cross-sectional study. *Am J Otolaryngol* 2021; **42**: 103138. 34214774 10.1016/j.amjoto.2021.103138

[24] Marcelli V, Spadera L, De Bernardo E *et al.* Symptom improvement after transtympanic tube placement in Ménière’s disease: preliminary observations. *Acta Otorhinolaryngol Ital* 2021; **41**: 467–473. 34734583 10.14639/0392-100X-N1705 PMC8569656

[25] Montandon P, Guillemin P, Häusler R. Prevention of vertigo in Ménière’s syndrome by means of transtympanic ventilation tubes. *ORL* *J Otorhinolaryngol Relat Spec* 1988; **50**: 377–381. 3231460 10.1159/000276016

[26] Ogawa Y, Otsuka K, Hagiwara A *et al.* Clinical study of tympanostomy tube placement for patients with intractable Ménière’s disease. *J Laryngol Otol* 2015; **129**: 120–125. 25633256 10.1017/S0022215115000079

[27] Brinson GM, Chen DA, Arriaga MA. Endolymphatic mastoid shunt versus endolymphatic sac decompression for Ménière’s disease. *Otolaryngol Head Neck Surg* 2007; **136**: 415–421. 17321870 10.1016/j.otohns.2006.08.031

[28] Thomsen J, Bonding P, Birgit B. The non-specific effect of endolymphatic sac surgery in treatment of Meniere’s disease: a prospective, randomised controlled study comparing “classic” endolymphatic Sac surgery with the insertion of a ventilating tube in the tympanic membrane. *Acta Otolaryngol* 1998; **118**: 769–773. 9870617 10.1080/00016489850182413

[29] Welling DB, Nagaraja HN. Endolymphatic mastoid shunt: a reevaluation of efficacy. *Otolaryngol Head Neck Surg* 2000; **122**: 340–345. 10699806 10.1016/S0194-5998(00)70044-1

[30] Albu S, Babighian G, Amadori M, Trabalzini F. Endolymphatic sac surgery versus tenotomy of the stapedius and tensor tympani muscles in the management of patients with unilateral definite Meniere’s disease. *Eur Arch Otorhinolaryngol* 2015; **272**: 3645–3650. 25488280 10.1007/s00405-014-3428-1

[31] Loader B, Beicht D, Hamzavi J-S, Franz P. Tenotomy of the middle ear muscles causes a dramatic reduction in vertigo attacks and improves audiological function in definite Meniere’s disease. *Acta Otolaryngol* 2012; **132**: 491–497. 22201453 10.3109/00016489.2011.642815

[32] Reichmayr C, Sterrer E, Bachtiar A *et al.* Tenotomy of the middle ear muscles: an unknown surgical approach in Meniere’s disease. *Wien Klin Wochenschr* 2019; **131**: 87–91. 30421285 10.1007/s00508-018-1405-1

[33] Harcourt JP, Lambert A, Wong PY *et al.* Long-term follow-up of intratympanic methylprednisolone versus gentamicin in patients with unilateral Menière’s disease. *Otol Neurotol* 2019; **40**: 491–496. 30870364 10.1097/MAO.0000000000002108

[34] Patel M, Agarwal K, Arshad Q *et al.* Intratympanic methylprednisolone versus gentamicin in patients with unilateral Ménière’s disease: a randomised, double-blind, comparative effectiveness trial. *The Lancet* 2016; **388**: 2753–2762. 10.1016/S0140-6736(16)31461-1 27865535

